# Alexithymic Trait and Voluntary Control in Healthy Adults

**DOI:** 10.1371/journal.pone.0003702

**Published:** 2008-11-12

**Authors:** Xiaosi Gu, Xun Liu, Kevin G. Guise, John Fossella, Kai Wang, Jin Fan

**Affiliations:** 1 Department of Neuroscience, Mount Sinai School of Medicine, New York, New York, United States of America; 2 Department of Psychiatry, Mount Sinai School of Medicine, New York, New York, United States of America; 3 Anhui Medical University, Hefei, China; James Cook University, Australia

## Abstract

**Background:**

Alexithymia is a personality trait characterized by deficiency in understanding, processing, or describing emotions. Recent studies have revealed that alexithymia is associated with less activation of the anterior cingulate cortex, a brain region shown to play a role in cognitive and emotional processing. However, few studies have directly investigated the cognitive domain in relation to alexithymia to examine whether alexithymic trait is related to less efficient voluntary control.

**Methodology/ Principal Findings:**

We examined the relationship between alexithymic trait and voluntary control in a group of healthy volunteers. We used the 20-item Toronto Alexithymia Scale (TAS-20) to measure alexithymic trait. Additionally, we examined state and trait voluntary control using the revised Attention Network Test (ANT-R) and the Adult Temperament Questionnaire (ATQ), respectively. Alexithymic trait was positively correlated with the overall reaction time of the ANT-R, and negatively correlated with the Effortful Control factor of the ATQ.

**Conclusions/Significance:**

Our results suggest that alexithymic trait is associated with less efficient voluntary control.

## Introduction

Alexithymia was first introduced in the field of psychosomatic medicine and has recently been referred to as a personality trait characterized by a deficiency in the cognitive processing of emotions, namely, difficulties in identifying and communicating emotions, and externally-oriented thinking [1]–[4]. These characteristics reflect a disruption in the conscious experience of emotions [2]–[4]. Voluntary control, as an important aspect of consciousness and the source of attention, is critical for regulating mental computations including emotional processes. [5], [6]. However, this supervisory system is possibly severely impaired in individuals with high alexithymia [1], [2], [7], [8]. In addition to difficulties in recognizing and expressing emotions, these individuals often manifest flattened emotions at default, yet accompanied by random and abrupt emotional outbursts which they cannot interpret; they also have overcontrol of their internal needs, an exaggerated defensive system, and dysregulated autonomic responses such as increased heart rate to emotion-evoking stimuli, although always report less emotional experiences [7], [9], [10]. All these manifestations indicate a disconnection between the physiological responses and the voluntary control of emotions in alexithymia.

The notion that alexithymic trait is associated with voluntary control is derived from the aforementioned findings from psychosomatic medicine, and has been supported by experimental psychology studies carried out in nonclinical samples as well as neuroimaging findings. Behavioral studies have reported that alexithymic individuals are impaired in the cognitive processing of emotions [11]–[13]. One study used both the 20-item Toronto Alexithymia Scale (TAS-20) and the Level of Emotional Awareness Scale (LEAS) to assess alexithymic trait in a community sample of 380 subjects [11], and the participants were asked to identify emotions in a Perception of Affect Task (PAT). People with higher alexithymia scores had a decreased ability of recognizing both verbal and nonverbal emotions. Another study found that healthy adults with higher TAS-20 scores showed a diminished priming effect from contextual information to emotional words [12]. In other words, the presentation of an emotional context facilitated the processing of a related emotional word in a lesser extent in people with higher alexithymia scores than those with lower alexithymia scores. These studies clearly demonstrated that alexithymic trait is associated with the ability of the cognitive processing of emotions. As is mentioned before, although the top-down control of physiological responses are disrupted, autonomic responses *per se* are not impaired in people with high alexithymia [10], [14]. Therefore, alexithymia is viewed as “blindfeel”, the emotional equivalent of blindsight [4]. According to this thesis, alexithymia is a deficit in reaching the conscious awareness and in maintaining the voluntary control of emotions, rather than a disruption in the sensory/perceptual aspect of emotions. Alexithymic individuals can be emotionally aroused just as much as non-alexithymic individuals; however, they would report they do not feel anything or do not know how they feel, and consequently can not regulate their emotional states.

Neuroimaging studies have further supported this view by revealing that alexithymic trait is associated with a common neural substrate subserving voluntary control. Voluntary control is known to be implemented by a brain network including the anterior cingulate cortex (ACC), and other frontoparietal regions [5], [6]. The ACC subserves a wide range of high-level functions including executive control, error detection, reward, anticipation, and consciousness [5], [15], [16]. The ACC is consistently activated in situations where competing information needs to be processed [17]–[20]. When the ACC is lesioned, executive control is likely to be affected, although this is not always the case [21]–[23]. On the other hand, accumulating evidence also suggests that the ACC is associated with overall reaction time (RT) in cognitive tasks, which represents the general efficiency of voluntary control. fMRI and PET studies have reported that the amplitude of ACC activation changes as a function of RTs in various cognitive tasks [24], [25]. Decreased ACC volumes are associated with longer RTs in cognitive tasks where controlled processes are required [26]. Indeed patients with focal ACC lesion have slower response speed during cognitive tasks [22]. We expect that a normal individual with a less efficient ACC, in that case, would also have diminished executive control, or slower response speed in cognitively challenging tasks, or both.

Interestingly, a deficiency in the ACC is indeed evident in alexithymic individuals [2]–[4]. In a positron emission tomography (PET) study by Lane and colleagues, a group of healthy adults performed an emotion-generating task in the scanner and completed the LEAS [27]. Covariate analysis revealed a significant cluster of activity in the dorsal ACC (Brodmann's area 24) that was positively correlated with LEAS scores. A more recent PET study replicated these results, and further demonstrated that the correlation between emotional awareness and dorsal ACC activity was specific to highly arousing pictures, and was stronger in women than men [28]. Other functional magnetic resonance imaging (fMRI) and PET studies reported that alexithymic individuals had decreased activation of the dorsal ACC in response to painful pictures [29] and emotional movie clips [30]. In the “blindfeel” theory, Lane proposed clearly that the ACC might be the core neuroanatomical structure involved in alexithymia [4]. Together with the evidence indicating the role of the ACC in voluntary control, it is likely that a person with more profound alexithymic trait would also possess poorer capacity of voluntary control, because of the common cause of a less efficient ACC function.

To date, few studies have directly tapped the relationship between alexithymic trait and voluntary control in general, especially in healthy adults, although a few domains of cognitive functions have been studied in association with alexithymia in patients. This line of research is important in that a significant correlation between measurement of voluntary control and that of alexithymia could verify that the two constructs are behaviorally relevant; and that a nonclinical sample would better elucidate the nature of alexithymia as a personality trait and/or an endophenotype and exclude the confounding factors introduced by other neurological and psychiatric conditions in patient studies. More specifically, it remains unclear (1) whether this impairment is specific to emotional processing, or is also related to a deficit in general cognitive processing, or a result of interaction of both cognitive control and emotional processing, and (2) which aspects of voluntary control might be related to alexithymic trait.

We used the TAS-20 to measure alexithymic trait in a group of healthy adult participants. Additionally, we measured participants' performance on the revised Attention Network Test (ANT-R) (state voluntary control), and individual differences in effortful control using the Adult Temperament Questionnaire (ATQ) (trait voluntary control). The ANT-R is a relatively challenging version that measures the general efficiency of voluntary control of attention, and the three networks subserving attention (alerting, orienting, and executive control). Correlation and regression analyses were performed on TAS-20 scores and participants' performance on ANT-R, and ATQ scores, to examine the association between alexithymic trait and voluntary control, and other aspects of temperament. We hypothesized that high alexithymia is correlated with (1) lower efficiency of state voluntary control indicated by slower response speed on the ANT-R task, and greater conflict effect; and (2) a deficiency in trait voluntary control, indexed by low scores on ATQ subscale Effortful Control.

## Methods

### Participants

Thirty young healthy adult volunteers without reporting any neurological or psychiatric disorders (15 females and 15 males; mean age, 25.4 years; range, 22–34 years) participated in this study. The consent procedure was approved by the institutional review board and written informed consent was obtained from each participant.

### The 20-item Toronto Alexithymia Scale (TAS-20)

Alexithymic trait was measured by the TAS-20, which has been validated in both patient and nonclinical samples [31], [32]. The TAS-20 is a 20-item self-report questionnaire that was designed to measure the ability to regulate and communicate one's own emotions. The questionnaire is based on a 5-point scale from “1-strongly disagree” to “5-strongly agree”. A high score indicated high alexithymia – great difficulty in emotional awareness and regulation. The TAS-20 includes three factors: difficulty identifying feelings (DIF, e.g. “I am often confused about what emotion I am feeling”), difficulty describing feelings (DDF, e.g. “I am often confused about what emotion I am feeling”), and externally-oriented thinking (EOT, e.g. “I prefer to analyze problems rather than just describe them”). The factor structure of TAS-20 has been largely confirmed by confirmatory factor analysis (CFA) [31].

### The revised Attention Network Test (ANT-R)

We used two measurements to assess state and trait voluntary control, respectively: the revised Attentional Network Test (ANT-R), and the Adult Temperament Questionnaire (ATQ). ANT-R and the original version of Attentional Network Test (ANT) were designed to assess three attentional networks that are critically involved in voluntary control: *alerting*, which refers to the ability of achieving and maintaining a vigilant state; *orienting*, for the selection of certain information out of numerous incoming stimuli; and *executive control*, a more complex system that monitors and resolves conflicts between competing processes[5], [6]. The ANT-R is designed to increase the task difficulty and to challenge one's ability to rapidly and accurately process information, thus the overall RT is an index of the efficiency of mental operation under complex and unpredictable situations.

**Figure 1:**
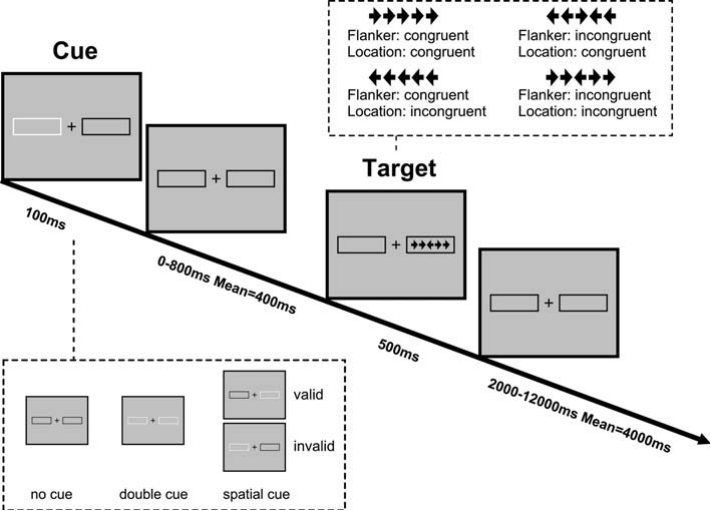
Schematic of the revised Attention Network Test (ANT-R). In each trial, depending on the cue condition (none, double, and valid or invalid cues), a cue box flashes for 100 ms. After a variable duration (0, 400, or 800 ms), the target (the center arrow) and two flanker arrows on the left and right side (congruent or incongruent flankers) are presented for 500 ms. The participant makes a response to the target's direction. The post-target fixation period varies between 2000 to 12000 ms.

The task is illustrated in Figure 1. Details of the task has been described elsewhere (Fan et al., under review). In brief, there are three cue conditions in each run: no-cue (baseline, 12 trials), double-cue (alerting, temporally informative, 12 trials), and spatial-cue (alerting and orienting, temporally and possibly spatially informative, 48 trials). RTs for the no- and double-cue conditions are used to assess the alerting benefit. To introduce the orienting component, a spatial cue and the subsequent stimulus are presented left or right of a fixation crosshair shown in the center of the screen. Participants need to shift their attention from the fixation point to the target stimulus to make the correct response. The validity of the spatial cue is manipulated in order to measure the disengagement and move operations (see [33], [34]. Specifically, 75% of the 48 spatial cues (36 trials) are valid and 25% (12 trials) are invalid. The probability of valid cue is the sum of the individual conditions of no-cue, double-cue, and invalid cue. Interpretation of these comparisons should be made with caution because of the frequency difference.

To introduce the conflict effect, the target (center arrow) is flanked on either side by two arrows of the same direction (congruent condition), or of the opposite direction (incongruent condition). To challenge the executive control function, double conflict that combines the flanker conflict effect [35] and the location conflict (Simon) effect [36] is introduced. There are 2 flanker congruency (congruent, incongruent) and 2 location congruency (congruent, incongruent) conditions.

A fixation cross is visible at the center of the screen throughout the duration of the task. In each trial, depending on the condition, either a transient cue (flashing of the box surrounding the stimulus row) is presented for 100 ms (the cued conditions) or the stimulus display remains unchanged (the no cue condition). After a variable duration (either 0, 400, or 800 ms, mean = 400 ms), the target and flankers are presented and remain visible for 500 ms. Cue-to-target interval is manipulated to challenge and measure the alerting and orienting speed. The duration between the offset of the target and the onset of the next trial is varied systematically, approximating an exponential distribution ranging with a mean trial duration of 5000 ms. The response collection window closes 1700 ms after the onset of the target and flankers as used in our original study [37]. The experiment consists of 4 runs, each with 72 test trials. The total duration for each run is 420 seconds. The total time required to complete this task is about 30 minutes.

The significant increase in attentional demands compared to our original design [37] is introduced by (1) manipulating the cue-to-target interval (0, 400, 800 ms), and using the brightening box for alerting as in a modified version of the ANT by Fernandez-Duque and Black [38]; (2) displaying the target on the left or right side of the fixation, manipulating cue validity to introduce the disengagement component, and extending the visual angle to create a larger size of the orienting effect; and (3) introducing the flanker by location dual conflict, and displaying the target only for 500 ms instead of 1700 ms.

The function of each of the three attentional networks is operationally defined as a comparison of the performance (RT and accuracy) between one condition and the appropriate reference condition, resulting in a score for each attentional network.

1. The phasic alerting (benefit) effect is defined as: *Alerting = RT _no cue_−RT _double cue_*, representing the benefit of the target response speed because of alerting.
2. Orienting operations can be separately measured as: *Validity effect = Disengaging+(Moving+Engaging) = RT _invalid cue_−RT _valid cue_*
*Moving+Engaging = RT _double cue_−RT _valid cue_*, for the benefit of target response under valid cue condition because of orienting and engaging in advance. Here, the Moving+Engaging is equivalent to the “orienting” effect we defined in our previous study [37].
*Disengaging = RT _invalid cue_−RT _double cue_* for the cost of disengaging from invalid cue.
  In addition, *Orienting time = RT _valid cue, 0 ms cue-to-target interval_−RT _valid cue, 800 ms cue-to-target interval_* for benefit of the target response because of the advanced orienting.
3. The conflict (cost) effect is defined as:
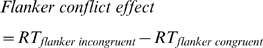


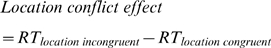

The inhibition of return (IOR) effect [33], [39] (if the difference is positive) or the cost of invalid cue under shorter (0 ms) compared to longer (400 ms) cue-target interval (if the difference is negative) is defined as: IOR = *(RT _invalid cue, 0 ms cue-to-target interval_−RT _valid cue, 0 ms cue-to-target interval_)−(RT _invalid cue, 400 ms cue-to-target interval_−RT _valid cue,400 ms cue-to-target interval_)*.

The effects in accuracy follow the same formulas. Here, the interactions between attentional networks were not specific defined because we did not have related hypotheses to test in this study.

The task was compiled and run on a PC, with a 17 inch LCD monitor, using E-Prime™ software (Psychology Software Tools, Pittsburgh, PA). Participants performed a brief practice task on a PC until they demonstrate at least 90% accuracy. Participants then performed the actual test.

### The Adult Temperament Questionnaire (ATQ)

We administered ATQ to measure trait voluntary control, considering there is strong evidence suggesting that alexithymia is largely inherited [40]–[43], and that ATQ includes an Effortful Control subscale that measures the ability of voluntary control derived from biology.

The long version ATQ is a self-report questionnaire of 177 items and four factors. The factors and facets of ATQ are: 1) Effortful Control (EC): Inhibitory Control, Activational Control, and Attentional Control; 2) Extraversion (E): Sociability, High Pleasure, and Positive Affect; 3) Negative Affect (NA): Fear, Sadness, Discomfort, and Frustration; and 4) Orienting Sensitivity (OS): Internal Perceptual Sensitivity, External Perceptual Sensitivity, and Affective Perceptual Sensitivity. These factors have been shown to be closely associated with the Big Five personality factors [44]. Scores range from 1 (“extremely untrue of you ”) to 7 (“extremely true of you”). The ATQ is a psychometrically sound instrument to measure temperament based on Derryberry and Rothbart's temperament model [45]. The validity and reliability of ATQ have been previously confirmed by factorial analysis [44].

### Statistical analysis

Means, standard deviations (SDs) were calculated for TAS-20, ANT-R, and ATQ. Skewness and kurtosis were also calculated to examine the distribution of these scores. Pearson correlation was used to examine the relations between TAS-20 and ANT-R, and between TAS-20 and ATQ. We further performed multiple regression analyses to examine the common and unique association among these variables. The first model included alerting, orienting, and flanker conflict scores as independent variables (IVs) and the overall score of TAS-20 as the dependent variable (DV). We then assessed a model where the four factors of ATQ were used as IVs and the overall score of TAS-20 as the DV. Finally we tested a model with the overall RT of ANT-R, the flanker conflict effect, and the EC factor of ATQ as IVs and TAS-20 overall score as the DV. The last model was used to examine the unique contribution of the general efficiency of state voluntary control (ANT-R overall RT) and trait voluntary control (EC).

## Results

### Alexithymic trait: Statistics and correlations of TAS-20

**Figure 2:**
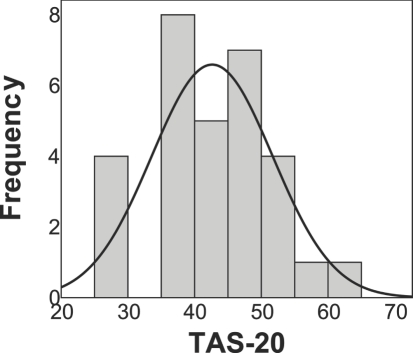
Distribution curve of the overall scores of TAS-20.

The mean scores, SDs, distribution, and correlations of TAS-20 and its three factors are listed in Table 1. The overall mean score was 42.6 (SD = 9.08, range 26–62), which is very close to the published norm [46]. This also indicates that on average our subject sample was not high alexithymia (cutoff point = 61), although one subject scored 62 and can be considered as high alexithymia [46]. The skewness and kurtosis of TAS-20 scores indicated that the distributions of TAS-20 scores were normal. We then performed a Shapiro-Wilk test [47] on these scores and the *p* values were all well above .05, indicating that the alexithymia scores in our subject sample were not deviated from normal distribution. The distribution curve of TAS-20 overall scores is shown in Figure 2. These results indicate that as a personality dimension, alexithymia is a continuous variable with normal distribution in healthy adults, which is consistent with previous findings [46], [48], [49].

**Table 1:** Means, SDs, distribution, and correlations of TAS-20 scores.

	DIF	DDF	EOT	Overall
***Descriptives***				
Mean	13.7	11.7	17.2	42.6
SD	3.74	3.81	4.00	9.08
Skewness	.35	.28	.39	−.10
Kurtosis	−.45	−.48	−.71	−.16
***Correlations***				
DDF	.55**			
EOT	.32	.41*		
Overall	.78**	.83**	.75**	

Note: * Correlation is significant at the 0.05 level (2-tailed).

** Correlation is significant at the 0.01 level (2-tailed). DIF: difficulty identifying feelings; DDF: difficulty describing feelings; EOT: externally – oriented thinking.

Table 1 also lists the correlations of TAS-20 and its subscales. Difficulty in Identifying Feelings (DIF) was significantly correlated with Difficulty in Describing Feelings (DDF) (*r* = .55, *p*<.01). DDF was also correlated with Externally-Oriented Thinking (EOT) (*r* = .41, *p*<.05). No significant correlation was found between DIF and EOT.

### Correlations between alexithymic trait and performance on ANT-R

The operationally defined effects of ANT-R are shown in Table 2. Error trials, which included incorrect and missing responses, were excluded from the calculation of mean RTs. We checked the distributions of the mean RTs to ensure the validity of correlation analysis. All the RTs were normally distributed in this sample. For the attentional networks, the *alerting* effect was 29±24 (mean±SD) ms. The *validity* effect was 95±32 ms. Breaking down the *orienting* effect, the *moving+engaging* effect was 41±21 ms, and the *disengaging* effect was 54±24 ms. The cost of invalid cue under 0 ms cue-target interval was −60±39 ms and the *orienting time* was 57±31 ms. The cost of invalid cue under 0 ms cue-target interval here is the cost under short cue-target interval and the “*orienting time*” is an index of the orienting cost in time. The *flanker conflict* effect was 137±43 ms and the *location conflict* effect was −11±27 ms. The negative value of the *location conflict* effect indicates that the RT was shorter under the location incongruent condition, indicating an opposite direction of the location conflict effect. The global mean RT of ANT-R, representing the overall efficiency of voluntary control, was 605±59 ms.

**Table 2:** Means and SDs of ANT-R, and their correlations with TAS-20.

	*ANT-R*		*TAS-20*			
*ANT-R*	*Mean*	*SD*	DIF	DDF	EOT	Overall
Alerting	29	24	−.17	.25	−0.15	−.03
Validity	95	32	−.14	.20	−.28	−.10
Moving+Engaging	41	21	−.21	−.01	−.29	−.20
Disengaging	54	24	.00	.28	−.13	.06
IOR	−60	39	−.04	−.29	−.02	−.15
Orienting	57	31	−.16	.27	−.20	−.04
Flanker conflict	137	43	.17	.11	−.05	.10
Location conflict	−10	27	−.10	−.03	−.04	−.07
Overall RT	605	59	.43*	.29	.17	.37*

Note: * Correlation is significant at the 0.05 level (2-tailed).

** Correlation is significant at the 0.01 level (2-tailed). DIF: difficulty identifying feelings; DDF: difficulty describing feelings; EOT: externally-oriented thinking; IOR: inhibition of return.

**Figure 3:**
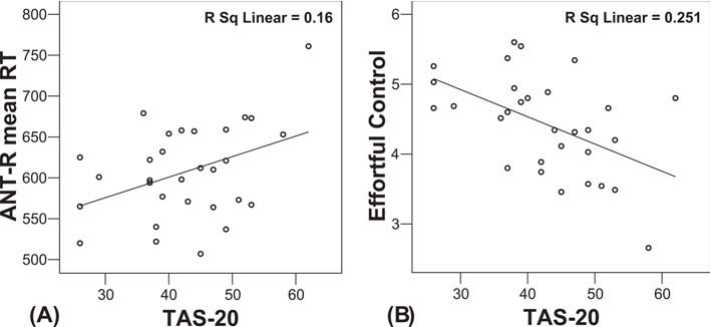
Correlations between overall TAS-20 score and (A) response speed of ANT-R, and (B) the Effortful Control subscale of ATQ.

The correlations between TAS-20 and ANT-R are also shown in Table 2. The overall score of TAS-20 was positively correlated with the mean RT of ANT-R (*r* = .37, *p*<.05, see Figure 3A), indicating that subjects who were more alexithymic responded more slowly on the ANT-R task. In addition, TAS-20 subscale DIF was correlated with overall RT of ANT-R (*r* = .43, *p*<.05). Surprisingly, no significant correlation was found between TAS-20 (overall and subscales) and any of the individual effects of ANT-R, including the conflict effect.

We also examined the overall response accuracy and the accuracy of each effect. There was no significant correlation between the global mean accuracy of ANT-R and TAS-20 overall score ( *r* = −.26, *p* = .16), or between the accuracy of any effect of ANT-R and TAS-20 scores, indicating there was no speed-accuracy tradeoff.

### Correlations between alexithymic trait and ATQ

Table 3 shows means and SDs of ATQ, and the correlations between TAS-20 and ATQ scores. We examined the distributions of the ATQ scores to ensure the validity of correlation analysis, and all the scores are normally distributed. As expected, participants who were more alexithymic scored lower on Effortful Control (*r* = −.50, *p*<.01, see Figure 3B), suggesting alexithymia is reliably correlated with trait voluntary control. Moreover, alexithymia was inversely correlated with Extraversion (*r* = −.41, *p*<.05), and positively with Negative Affect (*r* = .51, *p*<.01). No significant correlation was found between the overall score of TAS-20 and Orienting Sensitivity.

**Table 3:** Means and SDs of ATQ, and correlations between TAS-20 and ATQ scores.

	*TAS-20*					
*ATQ*	Mean	SD	DIF	DDF	EOT	Overall
**Effortful Control**						
Activation Control	4.6	.99	−.34	−.09	−.26	−.30
Attentional Control	4.1	.99	−.52**	−.37*	−.38*	−.53**
Inhibitory Control	4.6	.67	−.46**	−.14	−.22	−.35
**Overall**	4.4	.71	−.55**	−.26	−.37*	−.50**
**Extraversion**						
High Pleasure	4.5	.83	−.25	−.19	−.35	−.34
Positive Affect	5.0	.84	−.26	−.42*	−.45*	−.48**
Sociability	5.0	.94	−.09	−.40*	−.26	−.32
**Overall**	4.8	.82	−.21	−.37*	−.38*	−.41*
**Negative Affect**						
Discomfort	4.1	.86	.31	.21	.28	.34
Fear	4.2	.51	.49**	.37*	.32	.50**
Frustration	3.5	.59	.32	.19	.35	.37*
Sadness	4.0	.96	.43*	.25	−.03	.27
**Overall**	4.0	.54	.52**	.34	.34	.51**
**Orienting Sensitivity**						
Affective Perceptual Sensitivity	4.7	.67	−.19	−.16	−.18	−.22
Associative Sensitivity	4.5	.68	.15	.20	.19	.06
Neutral Perceptual Sensitivity	4.6	.79	.00	.03	.12	.07
Overall	4.6	.59	−.01	.03	−.09	−.03

Note: *Correlation is significant at the 0.05 level (2-tailed).

** Correlation is significant at the 0.01 level (2-tailed). DIF: difficulty identifying feelings; DDF: difficulty describing feelings; EOT: externally – oriented thinking.

The correlations between the three factors of TAS-20 and the factors and facets of ATQ are also listed in Table 3. DIF was negatively correlated with both Attentional Control and Inhibitory Control, indicating greater difficulty in identifying feelings is associated with reduced attentional and inhibitory control. DDF and EOT were only negatively correlated to Attentional Control. DDF was also negatively correlated to Positive Affect and Sociability, indicating increased difficulty in describing feelings was associated with decreased positive affect and sociability. EOT was also negatively correlated to Positive Affect. In addition, DIF was positively correlated to Fear and Sadnness, suggesting more difficulty in identifying feelings was related to more fear and sadness. DDF was also positively correlated to Fear. No significant correlation was found between the TAS-20 subscales and Orienting Sensitivity.

### Multiple regression models

We first examined a multiple regression model with alerting, orienting, and flanker conflict scores as IVs and TAS-20 overall score as the DV, and the model was not significant (*F*(3,26) = .10, *p*>.05).

A second multiple regression model with the four factors of ATQ (EC, E, NA, and OS) as IVs and the overall TAS-20 score as the DV was significant (*F*(4,25) = 4.14, *p* = .01), and explained 39.8% of the variance in TAS-20. However, only the contribution of Extraversion reached a marginal significance (E: β = −.37, *p* = .088). None of the other IVs made a significant contribution to TAS-20 in this model (EC: β = −.27, *p*>.05; NA: β = .23, *p*>.05; OS: β = .14, *p*>.05).

Lastly the overall RT of ANT-R, EC, and flanker conflict was entered together as IVs. The model was significant (*F*(3,26) = 4.41, *p* = .01), and explained 33.7% of the total variance in TAS-20. In this model, EC had a significant contribution to TAS-20 (β = −.45, *p* = .01); ANT-R mean RT reached a marginal significance (β = .30, *p* = .099); and the contribution of flanker conflict was still not significant (β = .003, *p*>.05). This model confirmed our findings from the correlation analyses that the overall efficiency of ANT-R and Effortful Control contributed significantly to alexithymic trait, while conflict processing did not yield similar effect.

## Discussion

The main finding of the current study is that alexithymic trait is closely related to the general efficiency of state voluntary control on a cognitive task, and trait voluntary control measured by the ATQ. Subjects with higher TAS-20 scores responded more slowly on the ANT-R, a task that requires rapid information processing, and scored lower on Effortful Control factor of the ATQ. In addition, alexithymic trait is associated with two other factors of ATQ, Extraversion and Negative Affect, indicating that alexithymic trait is a stable and inheritable.

An important finding from the ANT-R task is that alexithymic trait is closely related to the general efficiency of state voluntary control, rather than the individual attentional networks subserving voluntary control. This is in accordance with previous findings on the association between response efficiency and alexithymia characteristics in the context of emotional processing. For example, subjects with high TAS-20 scores displayed impaired task performance on a signal-detection paradigm only under a time constraint condition, but not under a temporally luxurious condition [50]. This suggests that alexithymia is a deficit in the fast processing of emotional information. Alexithymic individuals also had longer response latency in naming emotional words compared with controls [51]. Compared with previous studies, the ANT-R used in the current study does not involve any emotional valence, thus is an objective and direct measurement of the general efficiency of fast information processing and voluntary control. Our finding of a significant relationship between the overall score of TAS-20 and global mean RT of ANT-R suggests that the alexithymic characteristics are associated with less efficient voluntary control in general, instead of voluntary control of emotions *per se*.

Surprisingly, we did not find a significant relationship between the alexithymic trait and conflict processing. Conflict processing is an important component of voluntary control. It involves complex mental operations and is mostly used in conflict detection and conflict resolution. One would expect that conflict processing should be disrupted, if the overall efficiency of voluntary control is impaired. However, our data suggest that although the overall response speed is indeed slower in people with higher alexithymia scores, conflict processing is not necessarily disrupted in these individuals. This coincides with the finding that when the general slowness was taken into account, the significant greater conflict effect observed in patients with Alzheimer's disease disappeared [38]. Lesion studies have showed that patients with focal ACC lesion have normal conflict processing while performing Stroop or go-nogo tasks [22], [52], [53], but with a slower overall response speed. These studies may suggest that the general response speed and conflict processing are driven by a common factor. In the context of this study of healthy adults, less extreme TAS scores may not be sufficient to produce an impairment in conflict processing. Considering the limited sample size used in the current study, further investigation is needed to validate the association between alexithymia and executive control by preselecting and comparing groups with high and low alexithymia to increase the effect size.

The significant correlation between TAS-20 and the EC factor of the ATQ further demonstrated that alexithymic trait is also tightly related to trait voluntary control. Temperament is believed to be the part of personality that is biologically rooted [5], [44], [54]. Accordingly, temperamental effortful control refers to dispositional cognitive capacities that allow us to initiate or inhibit a particular response. These cognitive control capacities start to develop at early stages of life and are relatively stable across the lifespan [44]. If the general ability to initiate a response is impaired, it is reasonable to argue that the ability to produce a response to emotional stimuli will also be disrupted.

The association between alexithymic trait and voluntary control coincides with the role of the ACC in linking cognitive and emotional processes [15], [55]–[57]. Recent brain imaging studies have repeatedly reported ACC activation in emotional processes, especially in high-level voluntary processing of emotions rather than low-level responses to emotional stimuli [18], [27], [58]–[61]. More importantly, neuroimaging studies have showed abnormal activation of the ACC in individuals with high alexithymia [29], [30], [62]–[66]. A lesion study reported that a patient with a right anterior cingulate infarct presented with an alexithymia-like disorder [67]. Thus it is likely that alexithymic individuals have deficits in the ACC, supported by converging evidence from functional and structural neuroimaging studies and lesion studies.

A secondary aim of the current study was to examine the relationship between alexithymia and temperament. We found that alexithymia is associated with two temperament dimensions other than Effortful Control – Extraversion and Negative Affect. Previous studies have used other personality questionnaires including the NEO Five-Factor Inventory (NEO-FFI)[68], [69], and the Temperament and Character Inventory[70], [71]. These studies have consistently reported that high alexithymia is correlated with low extraversion/ openness and high neuroticism/negative emotions, which is consistent with our findings. The association between alexithymia and temperament indicates that alexithymia is a stable personal trait [70], [72], [73] that is largely derived from our temperament and biological endowments. This view can be further supported by behavioral genetics studies suggesting that alexithymia is largely influenced by genetic factors [40], [41], [43].

In summary, the current study suggests that more profound alexithymic trait is closely related to less efficient voluntary control. Alexithymic trait and voluntary control are behaviorally relevant and also possibly share the ACC as a common neural substrate. Alexithymic trait is also associated with less extraversion and more negative affect in our nonclinical sample. These findings may facilitate our understanding of the alexithymic trait construct and may have theoretical implications for future research on its neural basis
